# Evaluation of the Choroidal Thickness and Retinal Nerve Fiber Layer Thickness in Patients with Vasovagal Syncope

**DOI:** 10.3390/jpm15060259

**Published:** 2025-06-18

**Authors:** Hasan B. Isleyen, Batur G. Kanar, Guzide Akcay, Serdar Demir, Hatice S. Kanar, Mehmet V. Yazicioglu

**Affiliations:** 1 Cardiology Department, Istanbul Nişantaşı Üniversitesi, Istanbul 34475, Turkey; isleyenburak44@gmail.com; 2 Cardiology Department, School of Medicine, Istinye University, Istanbul 34396, Turkey; mvefik@yahoo.com; 3 Ophthalmology Department, Dr Lütfi Kırdar Kartal Eğitim ve Araştırma Hastanesi, Istanbul 34862, Turkeyhselensonmez@hotmail.com (H.S.K.); 4 Cardiology Department, Kartal Koşuyolu High Specialization Training and Research Hospital, Istanbul 34846, Turkey; sdemirmd@gmail.com

**Keywords:** choroidal thickness, optical coherence tomography, vasovagal syncope

## Abstract

**Aim**: The aim of this study was to evaluate choroidal and peripapillary retinal nerve fiber layer (RNFL) thicknesses in individuals with vasovagal syncope (VVS). **Method**: A total of 67 consecutive patients with VVS and 61 healthy control subjects were enrolled this study. The choroidal thickness (CT) at the fovea, the nasal to fovea thickness, and the temporal to fovea thickness were measured, alongside pRNLFT measurements assessed by swept-source optical coherence tomography (SS-OCT). **Results**: The mean foveal CT (408.7 ± 92.5 μm vs. 342.1 ± 60.2 μm, *p* < 0.01), the mean nasal CT (385.2 ± 88.3 μm vs. 329.2 ± 47.6 μm, *p* < 0.001), and the mean temporal CT (379.5 ± 51.6 μm vs. 321.48 ± 43.2 μm, *p* < 0.03) were statistically thicker in patients with VVS compared to the healthy controls. There was no statistically significant difference in the global pRNFLT measurements and all quadrants between the study groups. **Conclusions**: The CT in all regions was found to be thicker in patients with VVS compared to the healthy controls, while there were no differences in pRNFLT values. These results suggest that choroidal circulation might be affected by local neurotransmitter alterations in patients with VVS.

## 1. Introduction

Syncope is a loss of consciousness defined by a loss of postural tone, amnesia, and rarely, the disruption of smooth muscle tonus, with a short duration and spontaneous complete recovery [1]. Vasovagal syncope (VVS) is a common form of syncope that originates from a sudden failure of the autonomic nervous system to sustain adequate vascular tone under orthostatic stress. It may be a compensatory maneuver to protect the central nervous system and heart from permanent ischemic damage. Even though VVS is commonly benign, repeated episodes could adversely affect quality of life and might increase the risk of major adverse events [2].

In vasovagal syncope, the cardioinhibitory pathway involves parasympathetic hypertonicity, which affects the sinoatrial and atrioventricular nodes and may cause a long asystole or nodal rhythm [3]. On the other hand, the vasodepressor efferent pathway causes a decrease in vascular sympathetic tone, leads to arterial and venous dilation, and results in a reduction in peripheral vascular resistance and arterial blood pressure. Consequently, blood is pooled in the lower extremities and/or in the splanchnic beds, and venous return is reduced. As a result, stroke volume decreases, causing cerebral and systemic hypoperfusion [1,3,4].

As far as recent studies are concerned, the pathophysiology of VVS has still not been clearly clarified. It has been proposed that the development of VVS is associated with changes in hemodynamic parameters that result from variations in the plasma levels of vasoactive substances, such as nitric oxide, catecholamines, endothelin, prostanoids, or vasopressin, as part of the body’s response to orthostatic stress [1,5]. Moreover, paradoxical peripheral vasodilation caused by endothelial dysfunction might be important in improper excessive hypotension during orthostatic stress in individuals with VVS. Factors like inflammation and oxidative stress have also been associated with impaired endothelial function in these patients [5,6].

The choroid has a highly vascular structure, and choroidal blood flow is crucial for the health of the retina, supplying oxygen and nutrients to its outer layers [7]. Neurogenic control significantly influences choroidal blood flow through the autonomic nervous system [8]. Various factors, including neurotransmitters and local metabolic signals, modulate these neurogenic controls [9]. This regulation is vital during changes in light conditions or visual tasks, ensuring efficient nutrient delivery and waste removal from retinal tissues. As the use of optical coherence tomography (OCT) technology, especially swept-source optical coherence tomography (SS-OCT) technology, has increased, non-invasive detailed evaluation of choroidal and retinal tissues has become possible [10].

In this study, we aimed to investigate whether dysregulation of the parasympathetic and/or sympathetic nervous system affects ocular structural measurements by evaluating choroidal thickness (CT) and peripapillary retinal nerve fiber layer thickness (pRNFLT) in individuals with VVS.

## 2. Materials and Methods

### 2.1. Patient Population

The data used in this study were acquired prospectively. This study was conducted in accordance with the principles outlined in the Declaration of Helsinki and received approval from the institutional ethics committee. Patients aged between 18 and 60 years who exhibited a history of recurrent episodes of apparent syncope, having experienced more than two such occurrences in the past six months, were included in the study. The inclusion criteria included (1) a positive drug-free head-up tilt table test (HUTT), in which syncope was deemed to have reproduced spontaneous symptoms; (2) absence of left ventricular systolic dysfunction and valve insufficiency and/or stenosis over a mild degree (estimated ejection fraction > 50% using transthoracic echocardiography); (3) absence of obesity (body mass index < 30), hematologic or biochemical abnormalities, or use of drugs known to predispose to orthostatic hypotension. The exclusion criteria can be listed as (1) patients not meeting the above criteria; (2) individuals with a history of systemic diseases that might affect CT and pRNFLT, such as systemic arterial hypertension, diabetes mellitus, and rheumatologic diseases based on self-reported medical history; (3) eyes with high myopia (>6D); (4) advanced-level cataracts, age-related macular degeneration, or a history of retinal vascular disease; (5) patients with abnormal intraocular pressure (IOP), glaucoma, or ocular hypertension; (6) patients with a history of intraocular surgery and tachycardia or autonomic dysfunction associated with neurologic disorders including Parkinsonism, autonomic failure, peripheral neuropathy, or postural tachycardia syndrome.

### 2.2. Head-Up Tilt Table Testing Protocol

All patients stopped taking cardioactive medication for at least five half-lives prior to HUTT. Each patient was gently secured to the tilt table, ensuring appropriate positioning to allow for the use of a footboard for weight bearing. HUTT was performed in the morning after a 12 h overnight fasting period. A minimum 15 min equilibration time, with the patients resting in a supine position in a dark, quiet environment, was allowed before the commencement of baseline recordings for the HUTT procedure. In our standard protocol, there were 15 min of rest followed by 45 min (or until the onset of symptoms) at a 70-degree angle [11]. The predictive value of HUTT was enhanced with sublingual glyceryl trinitrate [11,12]. Systemic arterial blood pressure and heart rate were continuously recorded, automatically calculating all the R-R intervals throughout the test using a non-invasive, beat-by-beat monitor CardioLab^®^ (GE Healthcare, Chicago, IL, USA), and data were collected by a computer that processed them using ad hoc software.

### 2.3. Ophthalmic Examinations

All participants in the study underwent an extensive and thorough ophthalmic assessment, which included a variety of tests designed to evaluate different aspects of visual function and ocular health. The evaluation began with an assessment of best-corrected visual acuity, which was performed using the Snellen chart. Additionally, intraocular pressure was measured employing the Goldmann applanation tonometry technique, a reliable method for gauging the pressure within the eye.

The examination also encompassed slit lamp biomicroscopy, a diagnostic procedure that allows for detailed observation of the anterior segment of the eye, and a dilated fundus examination to facilitate a comprehensive view of the retina and other posterior structures. To measure the axial length of the eye, the IOL Master 500 (manufactured by Carl Zeiss Meditec Inc., based in Jena, Germany) was utilized, providing precise measurements essential for evaluating ocular dimensions.

Furthermore, the choroidal thickness (CT) and the peripapillary retinal nerve fiber layer thickness (pRNFLT) were quantified using the swept-source optical coherence tomography (SS-OCT) system, specifically the Topcon Triton model from Japan. It is worth mentioning that all measurements and assessments were consistently carried out by the same trained technician during similar time frames, specifically between 9:00 a.m. and 12:00 p.m., to minimize potential variabilities. HUTT and OCT procedures were not performed on the same day.

In determining the choroidal thickness, which was defined as the axial distance from the retinal pigment epithelium (RPE) to the outer interface of the choroid/sclera, evaluations were conducted by a single ophthalmologist (H.S.K). When H.S.K performed the OCT evaluation, she did not know whether the patient had VVS or not, and the study was single-blind. The CT measurements were specifically taken in the subfoveal region, as well as at distances of 1500 μm temporally and 1500 μm nasally from the foveal region. For the assessment of the pRNFLT, the measurements were obtained using OCT by employing a circular scan with a diameter of 3.46 mm that was centered on the optic disk.

**Figure 1:**
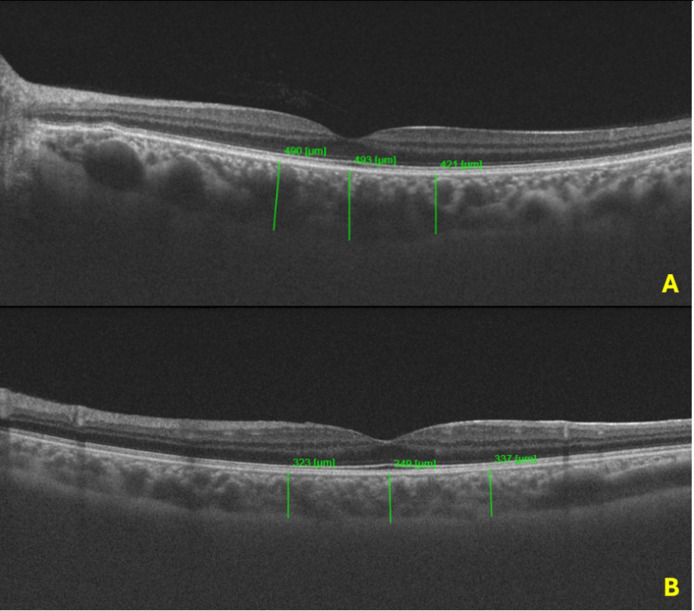
(**A**): Increased CT in the fovea, temporal to fovea, and nasal to fovea regions in a patient with VVS; (**B**): CT in the fovea, temporal to fovea, and nasal to fovea regions in a healthy control.

**Figure 2:**
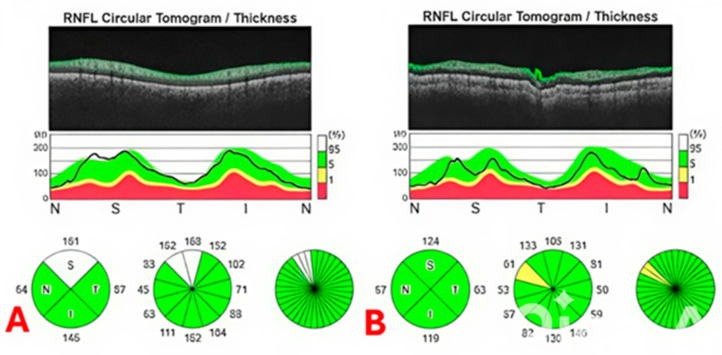
(**A**): pRNFL images of a healthy control; (**B**): pRNFL images of a patient with VVS.

The resulting data provided pRNFLT values for each of the four quadrants—superior (S), inferior (I), nasal (N), and temporal (T)—as well as the global mean values by using software program. It is important to note that the statistical analyses were conducted using the average values derived from both eyes of each participant, allowing for a comprehensive evaluation of the visual and ocular health indicators across the sample. Figure 1 and Figure 2 demonstrate the OCT and pRNLFT measurements for participants with VVS and a healthy control.

### 2.4. Statistical Analysis

Statistical analyses were conducted utilizing SPSS software, specifically version 21, provided by SPSS Inc., located in Chicago, IL, USA. Continuous variables in the study were presented as mean values accompanied by their corresponding standard deviation (SD). To ensure the validity of the assumed linear relationships and the principle of homoscedasticity across the datasets, we evaluated these assumptions through the examination of standardized residuals plots. Furthermore, the assumption of normality for the dependent variable was assessed using the Kolmogorov–Smirnov statistical criterion.

In addition, to detect significant differences among categorical variables, the Chi-square test was employed. As all continuous variables demonstrated a normal distribution, statistical comparisons of quantitative data were carried out using the unpaired-sample *t*-test method. It is important to note that differences were regarded as statistically significant when the *p*-value was less than 0.05. This level of significance indicates that we could reject the null hypothesis in favor of the alternative hypothesis, suggesting that there is a meaningful difference worthy of further discussion and analysis.

## 3. Results

A total of 67 patients with VVS were included in this study, and their data were compared with those of 61 age- and sex-matched healthy controls. The mean age of patients with VVS was 35.2 ± 6.3 years, while that of the control group subjects was 36.3 ± 7.4 years. Demographic and clinical information about the patients and control subjects is summarized in Table 1. No significant differences were noted in the values of intraocular pressure, axial length, systemic arterial blood pressure measurements, smoking status, age, and sex between patients with VVS and the healthy controls.

In the CT evaluation, a statistically significant difference was noted between patients with VVS and the healthy controls. The mean foveal CT (408.7 ± 92.5 μm vs. 342.1 ± 60.2 μm, *p* < 0.001), mean nasal CT (385.2 ± 88.3 μm vs. 329.2 ± 47.6 μm, *p* < 0.001), and mean temporal CT (379.5 ± 91.6 μm vs. 321.48 ± 43.2 μm, *p* < 0.001) were statistically thicker in patients with VVS compared to the healthy controls. There was no statistically significant difference in pRNFLT measurements between patients with VVS and the healthy controls. Table 2 summarizes the comparison of SS-OCT measurements between the study groups.

## 4. Discussion

In this study, we evaluated the CTs and pRNFLT in participants with VVS and compared these values with those of healthy controls. The main outcomes of this study are as follows: (1) the mean CT in all regions was significantly thicker in participants with VVS compared to the control group. (2) There were no significant differences in all pRNFLT measurements between study groups.

Both sympathetic and parasympathetic pathways contribute to choroidal blood flow regulation, with sympathetic fibers generally causing vasoconstriction and parasympathetic fibers promoting vasodilation [7]. Parasympathetic stimulation, particularly through acetylcholine release, promotes vasodilation, increasing blood flow to meet the metabolic demands of the retina, especially during high visual activity. In contrast, sympathetic activation, associated with norepinephrine, causes vasoconstriction, reducing blood flow [13]. Various neurotransmitters also influence choroidal blood flow regulation. For example, nitric oxide promotes vasodilation, while other molecules like vasoactive intestinal peptide and substance *p* additionally modulate vessel diameter [7,13].

The dysregulation of the autonomous nerve system and the alteration of neurotransmitters have crucial roles in VVS pathogenesis. Jardine at el. showed parasympathetic hypertonicity in participants with VVS [14]. Aksu et al. showed that low endothelin-1 levels, which is a pro-inflammatory vasoconstrictor peptide, are associated with a higher risk of VVS. In addition, neuropeptide Y may also play a role in VVS by increasing peripheral vascular resistance and decreasing cardiac output during orthostatic regulation [15]. Liao et al. revealed that neuropeptide Y plasma levels are significantly reduced in individuals with VVS [16]. Gallegos et al. showed that nitric oxide levels are increased in VVS, probably due to high catecholamine plasma levels. Nitric oxide synthase could be activated by β−2 receptors, increasing the release of nitric oxide from the vascular endothelium and leading to excessive vasodilation [17]. Endothelin-1 was found to be negatively correlated with choroidal blood flow, and endothelin-1 increases vasoconstriction in choroid and retinal blood. [18,19]. Some studies conducted with animals have shown that choroidal vasodilation and choroidal thickness may increase due to increased nitric oxide [20,21]. In this study, we found that the CT in the fovea, nasal to the fovea, and temporal to the fovea regions was statistically significantly thicker than in the healthy controls. Increased CT in participants with VVS might be related to the increase in parasympathetic activity and the change in neurotransmitters.

Thicker CT is linked to several ocular diseases and serves as an important biomarker for diagnosis and management [22,23]. Pachychoroid spectrum diseases, age-related macular degeneration, and some uveitic diseases are among the main diseases in which CT is important [22,24]. Patients with central serous chorioretinopathy have an especially high CT and type A personality traits [25,26,27]. It is known from previous studies that participants with VVS have stressed personality structures [28,29]. Therefore, participants with VVS may also be prone to ocular diseases such as central serous chorioretinopathy due to their high CT. Further studies may clarify this relationship.

The pRNFL is the region surrounding the optic disk where nerve fibers from the retina converge to form the optic nerve and is supplied by the central retinal artery [30]. This layer consists primarily of the axons of ganglion cells and is essential for transmitting visual information from the retina to the brain. The health of the pRNFL is vital for visual function, as changes in its thickness can indicate various ocular diseases, especially glaucoma [31,32]. Thinning of the pRNFL is often a sign of damage to the optic nerve and progressive loss of ganglion cells, necessitating careful monitoring [31]. We compared the pRNFLT values of participants with VVS with healthy controls, and we did not find any differences between participants with VVS and the healthy controls. It is known that there are distinct differences between choroidal and retinal artery blood flow [33]. Retinal blood flow is tightly regulated by local metabolic demands, allowing it to adjust to the oxygen needs of the inner retina. In contrast, choroidal blood flow is influenced more by systemic factors rather than local needs, leading to less autoregulation [33,34,35]. For this reason, while we detected changes in CTs in participants with VVS, we may not have detected changes in pRNFLT values, which indicate retinal artery blood flow.

This study has some limitations. For instance, the size of the study groups was relatively small. Moreover, a manual method was used to measure CTs instead of using an automated software program, which is more reliable. We evaluated only choroidal thickness and no other parameters such as the choroidal vascular index [36]. Patients’ anxiety status and/or personality evaluation were not recorded. In this study, we did not evaluate the neurotransmitter levels in plasma and/or serum. In the HUTT protocol, we did not use the Vasovagal Syncope International Study (BASIS) criteria for the subgroup analysis of the participants with VVS. The VASIS classification includes the following categories: mixed (VASIS-1), cardioinhibition (VASIS-2A), severe cardioinhibition/asystole (VASIS-2B), and pure vasodepression (VASIS-3). There may be differences in the subgroup analysis of the participants in terms of CT measurements [37].

## 5. Conclusions

In conclusion, we showed that participants with VVS exhibited greater CT across all measured regions when compared to healthy controls. Conversely, there were no significant variations observed in the thickness of the pRNFL. These findings imply that the choroidal blood flow could be influenced by changes in local neurotransmitter levels in individuals suffering from VVS. This interplay underscores the connection between cardiovascular health and CT alterations, suggesting a need for monitoring in individuals with frequent syncopal episodes to prevent complications and maintain ocular health. Further investigation into these dynamics may yield insights beneficial for clinical practice regarding VVS and ocular health.

## Figures and Tables

**Table 1:** Demographic and clinical data of the study population.

	Patients with VVS (*n* = 67)	Healthy Controls (*n* = 61)	*p* Value
Age (years)	35.2 ± 6.3	36.3 ± 7.4	0.13
Gender (Female%)	42(62.7%)	35(57.4%)	0.08
Body mass index (kg/m^2^)	22.4 ± 5.6	21.5 ± 3.9	0.23
Smoking	14(20.9%)	12(19.6%)	0.47
SRE (D)	+0.71 ± 0.2	+0.58 ± 0.3	0.55
Axial length (mm)	23.08 ± 0.4	22.98 ± 0.3	0.67
Intraocular pressure (mm Hg)	16.25 ± 5.3	18.04 ± 4.8	0.25
Systolic BP (mmHg)	108.3 ± 7.2	123.4 ± 9.3	0.22
Diastolic BP (mmHg)	76.7 ± 4.7	79.9 ± 5.9	0.12
Mean BP (mmHg)	86.7 ± 5.5	94.3 ± 6.9	0.19

Note: data are presented as mean ± standard deviation while categorical variables are expressed as percentages. Abbreviations: BP: blood pressure; SRE: spherical refractive error.

**Table 2:** Summary of statistical analyses for comparison of SFCT, pRNFLT (average), and quadrants of pRNFLT.

	Participants with VVS (*n* = 67)	Healthy Controls (*n* = 61)	*p* Value
Foveal CT (μm)	408.7 ± 92.5	342.1 ± 60.2	**<0.001**
Nasal CT (μm)	385.2 ± 98.3	329.2 ± 47.6	**<0.001**
Temporal CT (μm)	379.5 ± 91.6	321.48 ± 43.2	**<0.001**
Global pRNFLT (μm)	106.7 ± 7.4	108.2 ± 6.2	0.11
Inferior pRNFLT (μm)	128.6 ± 8.3	129.2 ± 8.9	0.41
Superior pRNFLT (μm)	127.6 ± 7.9	129.8 ± 8.4	0.31
Nasal pRNFLT (μm)	86.5 ± 6.7	87.6 ± 6.3	0.24
Temporal pRNFLT (μm)	84.4 ± 6.5	86.2 ± 6.8	0.26

Note: data are presented as mean ± standard deviation. Bold values indicate statistical significance *p* < 0.05. Abbreviations: CT: choroidal thickness; pRNFLT: peripapillary retinal nerve fiber layer thickness; SFCT: subfoveal choroidal thickness; VVS: vasovagal syncope.

## Data Availability

Certain confidentiality constraints prevent us from disclosing the underlying database used in this study.
